# Cognitive Functioning, Gender, and Marital Quality Among Older Married Couples: A Dyadic Approach

**DOI:** 10.1093/geroni/igaa057.2042

**Published:** 2020-12-16

**Authors:** Elizabeth Gallagher, Jeffrey Stokes

**Affiliations:** 1 University of Massachusetts Boston, Kingston, Massachusetts, United States; 2 University of Massachusetts Boston, Boston, Massachusetts, United States

## Abstract

Older spouses influence one another in myriad ways, and dyadic effects of marital quality on health and well-being have been well-established. However, little attention has been paid to dyadic implications of cognitive functioning, including for spouses’ perceptions of the relationship itself. This study examines associations of older husbands’ and wives’ cognitive functioning with both partners’ reports of four marital quality outcomes. Structural equation modeling analyzed data from 1,414 opposite-sex couples drawn from the 2016 wave of the Health and Retirement Study. Findings revealed that (a) wives’ poorer cognitive functioning was associated with wives’ reporting greater closeness and higher ratings of enjoying time with a spouse, whereas (b) husbands’ poorer cognitive functioning was associated with wives’ reporting greater marital strain, lower marital support, lower closeness, and lower ratings of enjoying time with a spouse. This suggests that cognitive functioning/impairment has dyadic consequences for marital quality, which are highly gendered.

